# Construction and validation of a predictive model for recurrent wheezing risk within one year after initial hospitalization for RSV lower respiratory tract infection in preschool children

**DOI:** 10.3389/fped.2026.1930501

**Published:** 2026-09-09

**Authors:** Yiyang Liu, Yuanshuai Fu, Yunqian Chi, Zihan Zhang, Yunhe Wang, Wei Hao

**Affiliations:** 1 School of Clinical Medicine, Shandong Second Medical University, Weifang, Shandong, China; 2 Department of Pediatrics, The First Affiliated Hospital of Shandong First Medical University & Shandong Provincial Qianfoshan Hospital, Jinan, Shandong, China

**Keywords:** 25-hydroxyvitamin D3, eosinophils, preschool children, recurrent wheezing, respiratory syncytial virus, risk prediction

## Abstract

**Background:**

Respiratory syncytial virus (RSV) is a leading cause of lower respiratory tract infection (LRTI) in preschool children and is strongly associated with subsequent recurrent wheezing; however, predictive tools for identifying high-risk children are lacking. This study aimed to construct and validate a model for predicting recurrent wheezing within one year after initial hospitalization for RSV LRTI.

**Methods:**

This retrospective study included preschool children with wheezing hospitalized for their first RSV LRTI at our hospital between April 2020 and March 2025. Patients were divided into recurrence and non-recurrence groups based on recurrent wheezing within one year post-discharge. Demographic and clinical data, and serum biomarkers (25-hydroxyvitamin D3 [25-(OH) D3], interleukin-6 [IL-6], interferon-*γ* [IFN-*γ*], eosinophil percentage, and other markers) were collected at admission.

**Results:**

A total of 480 patients were included, with 276 experiencing recurrence and 204 without. Multivariate analysis identified independent predictors: younger age (OR = 0.398, *P* = 0.007), passive smoking (OR = 2.802, *P* = 0.013), longer hospital stay (OR = 1.264, *P* = 0.003), lower 25-(OH) D3 (OR = 0.766, *P* < 0.001), higher IL-6 (OR = 1.591, *P* < 0.001), lower IFN-*γ* (OR = 0.563, *P* < 0.001), and higher eosinophil percentage (OR = 1.398, *P* < 0.001). The nomogram incorporating these factors achieved an AUC of 0.950 (sensitivity 0.912, specificity 0.857) in the training set and 0.940 (sensitivity 0.874, specificity 0.882) in the validation set.

**Conclusion:**

This nomogram, incorporating age, passive smoking, hospital stay, 25-(OH) D3, IL-6, IFN-*γ*, and eosinophil percentage, accurately predicts a recurrent wheezing risk and can guide personalized management for preschool children after RSV LRTI.

## 1 Introduction

Respiratory syncytial virus (RSV) is the leading cause of lower respiratory tract infections (LRTI) in preschool children, typically manifesting as bronchiolitis or pneumonia, accompanied by wheezing, coughing, and respiratory distress (1, 2). Although antiviral and anti-inflammatory treatments are available, management largely remains supportive, with no specific measures reliably preventing subsequent recurrent wheezing or asthma development (3, 4). A key clinical gap is the lack of effective tools to identify children at high risk of recurrent wheezing following severe RSV infection (5). This study aims to bridge this gap by developing a predictive model for recurrent wheezing within one year after the first hospitalization for RSV LRTI.

Increasing evidence suggests that RSV -induced airway inflammation involves complex immune mechanisms. An imbalance in the T helper cell type 1/T helper cell type 2 (Th1/Th2) balance, particularly alterations in Interleukin (IL)-6, Interferon-gamma (IFN-*γ*), and vitamin D levels, is associated with the development of post-viral recurrent wheezing (6, 7). Vitamin D deficiency, indicated by low serum 25-hydroxyvitamin D3 [25-(OH) D3] levels and reduced IFN-*γ* levels, may impair antiviral defenses, favoring Th2-driven eosinophilic inflammation (8, 9). While elevated IL-6 can perpetuate airway injury and remodeling (10). These immune changes, along with early exposures such as passive smoking and preterm birth, are thought to contribute to the development of a chronic wheezing phenotype (11, 12). Eosinophilic inflammation and impaired lung function can serve as early biomarkers for predicting subsequent recurrent wheezing, although their combined predictive value has not been fully explored (13).

This study aims to construct and validate a predictive model for assessing the risk of recurrent wheezing within one year after the first hospitalization for RSV LRTI in preschool children. The novelty lies in combining immune, inflammatory, and functional markers with environmental factors to create a practical bedside tool. This work provides clinicians with a practical tool for risk stratification and personalized management strategies for high-risk pediatric patients following RSV infection.

## 2 Materials and methods

### 2.1 Research subject

A retrospective analysis was conducted on 480 pediatric patients with wheezing and RSV LRTI admitted to our hospital from April 2020 to March 2025.The retrospective study was approved by the Ethics Committee of the First Affiliated Hospital of Shandong First Medical University & Shandong Provincial Qianfoshan Hospital (Approval Number: 2026-S687). Patient data were anonymized, and the requirement for written informed consent was waived due to the retrospective design of the study.

Inclusion Criteria: (1) Preschool children aged 3–6 years; (2) Wheezing diagnosis in accordance with the “Evidence-based guidelines for clinical practice in the diagnosis, treatment, management, and prevention of recurrent wheezing in infants and toddlers (2025)” (14); (3) First hospitalization due to RSV-induced LRTI; (4) Complete clinical information.

Exclusion Criteria: (1) Wheezing episodes caused by other identifiable causes (tracheomalacia, foreign body aspiration, gastroesophageal reflux, persistent bacterial bronchitis, etc.); (2) Pediatric patients with concurrent infections by other respiratory pathogens; (3) Pediatric patients with underlying diseases such as congenital heart disease, bronchopulmonary dysplasia, immunodeficiency disorders, or malignant tumors; (4) Pediatric patients lost to follow-up within one year after hospitalization.

### 2.2 Research methodology

(1) RSV Infection Determination and Treatment: Respiratory samples were collected from the pediatric patients. Real-time polymerase chain reaction (RT-PCR) (ABI 7500 FastDX, Applied Biosystems, USA) was used to detect the presence of human respiratory syncytial virus nucleic acid (15). A positive result indicated RSV infection. Infected patients received antiviral, anti-allergy, and anti-inflammatory treatments as symptomatic and supportive care.
(2) Criteria for Assessing Recurrent Wheezing: Wheezing was defined as a high-pitched whistling sound during exhalation, objectively confirmed by a clinician through auscultation during acute episodes. During scheduled telephone follow-ups, parents were questioned about respiratory symptoms; however, telephone reports alone did not qualify as confirmed wheezing events. Whenever a parent reported suspected wheezing or noisy breathing, the child was instructed to attend an in-person evaluation at our outpatient clinic or a local healthcare facility. Only episodes with documented auscultatory confirmation by a clinician (recorded in the electronic medical record) were counted toward the definition of recurrent wheezing. Regular follow-up contacts (telephone or outpatient visits) were routine clinical practice at 1, 3, 6, 9, and 12 months post-discharge. For this retrospective study, we reviewed these existing records to ascertain the number of clinician-confirmed wheezing episodes. If a patient experienced ≥2 wheezing episodes within 3 months, each lasting more than 24 h and separated by at least a 7-day symptom-free period, or had ≥3 wheezing episodes confirmed by a clinician within 12 months post-discharge, they were considered to have recurrent wheezing. Wheezing events clearly attributable to other causes (e.g., foreign body aspiration, acute exacerbation of gastroesophageal reflux, tracheomalacia, etc.) identified during follow-up were excluded from the count of recurrent wheezing. Because this was a retrospective study, exclusion was based strictly on objective documentation in the electronic medical record, including bronchoscopy findings, imaging evidence [chest x-ray or computed tomography (CT)], esophageal pH monitoring, or a definitive diagnosis explicitly recorded by the attending clinician. In cases where the medical record was inconclusive or the alternative etiology was merely suspected without confirmatory testing, the wheezing event was not excluded and was conservatively counted toward recurrent wheezing to minimize information bias.

Based on whether recurrent wheezing recurred within one year after hospitalization, the 480 pediatric patients were categorized into a non-recurrence group (*n* = 204) and a recurrence group (*n* = 276). In chronological order of hospitalization, 480 pediatric patients were divided into a training set (*n* = 301) and a validation set (*n* = 179). The training set included 127 non-recurrence patients and 174 recurrence patients, while the validation set included 77 non-recurrence patients and 102 recurrence patients.

### 2.3 Data collection

Retrospective data were collected through our hospital's electronic medical record system, including demographic and clinical factors, and serum markers at the time of admission. Demographic and clinical factors included gender, age, body mass index (BMI), place of residence, whether the child was preterm, history of allergies, family history of asthma, exposure to passive smoking, and length of hospital stay. For serum markers, the serum 25-(OH) D3 and IL-6 levels were measured using a fully automated electrochemiluminescence immunoassay analyzer (Cobas e601, Roche Diagnostics, Switzerland). C-reactive protein (CRP) was measured using a fully automated biochemical analyzer (AU5800, Beckman Coulter, USA). Serum interleukin IL-2, IL-4, and INF-*γ*, were detected using ELISA kits (SPS-11282, SPS-11285, SPS-11159, Shanghai SaipeiSen Biotechnology Co., Ltd., China). White blood cell count (WBC), eosinophil percentage, and neutrophil percentage were measured using a fully automated hematology analyzer (XN-1800, Sysmex, Japan). Complete blood count (including WBC, eosinophil percentage, and neutrophil percentage) and CRP are routine admission tests for all children with LRTI at our hospital. In addition, since 2020, our department has implemented a standardized clinical care protocol for RSV LRTI that routinely includes serum 25-(OH) D3, IL-6, and IFN-*γ* measurements as part of a comprehensive nutritional-immunological assessment to guide post-discharge management. This institutional practice ensured complete data availability for all biomarkers in the present cohort.

### 2.4 Statistical method

This study utilized R 4.3.3 software for data analysis. Categorical data were presented as n (%), and intergroup comparisons were conducted using the *χ*^2^ test. Continuous data were expressed as x ± s, and comparisons between two groups were performed using independent samples t-tests. Recurrent wheezing within one year after hospitalization (recurrence = 1, non-recurrence = 0) was used as the dependent variable in both univariable and multivariable logistic regression analyses to identify independent risk factors for recurrent wheezing within one year after initial RSV LRTI hospitalization. Receiver operating characteristic (ROC) curves were plotted to analyze the predictive value of each risk factor for recurrent wheezing within one year after initial RSV LRTI hospitalization. Independent predictors were selected to construct a nomogram prediction model for recurrent wheezing risk within one year after initial RSV LRTI hospitalization. The model's performance was evaluated through ROC curves and internal validation. A *P*-value < 0.05 was considered statistically significant.

## 3 Results

### 3.1 Training set

#### 3.1.1 Demographic and clinical factors in the training set

The demographic and clinical characteristics of the training cohort are displayed in Table 1. Gender, BMI, and place of residence did not differ significantly between groups (all *P* > 0.05). Compared with children who remained free of recurrence, those in the recurrence group were significantly younger (*P* < 0.001) and exhibited markedly higher proportions of preterm birth (*P* = 0.036), personal history of allergies (*P* = 0.016), family history of asthma (*P* = 0.004), and exposure to passive smoking (*P* = 0.028). The length of hospital stay was also significantly prolonged in the recurrence group (*P* < 0.001).

**Table 1:** Demographic and clinical factors in the training set.

Variables	Non-recurrence group (*n* = 127)	Recurrence group (*n* = 174)	t/*χ*^2^	P
Gender, n(%)			0.153	0.696
Male	76 (59.84%)	108 (62.07%)		
Female	51 (40.16%)	66 (37.93%)		
Age (years)	4.58 ± 0.64	4.25 ± 0.51	4.763	<0.001
BMI (kg/m^2^)	16.52 ± 2.37	16.24 ± 2.59	0.952	0.342
Place of residence, n(%)			0.043	0.835
Urban	70 (55.12%)	98 (56.32%)		
Country	57 (44.88%)	76 (43.68%)		
Preterm infant, n(%)			4.374	0.036
Yes	39 (30.71%)	74 (42.53%)		
No	88 (69.29%)	100 (57.47%)		
History of allergies, n(%)			5.839	0.016
Yes	80 (62.99%)	132 (75.86%)		
No	47 (37.01%)	42 (24.14%)		
Family history of asthma, n(%)			8.383	0.004
Yes	53 (41.73%)	102 (58.62%)		
No	74 (58.27%)	72 (41.38%)		
Exposure to passive smoking, n(%)			4.833	0.028
Yes	41 (32.28%)	78 (44.83%)		
No	86 (67.72%)	96 (55.17%)		
Length of hospital stay (d)	6.32 ± 2.15	7.84 ± 2.67	5.476	<0.001

BMI, body mass index.

#### 3.1.2 Serum markers in the training set

**Figure 1:**
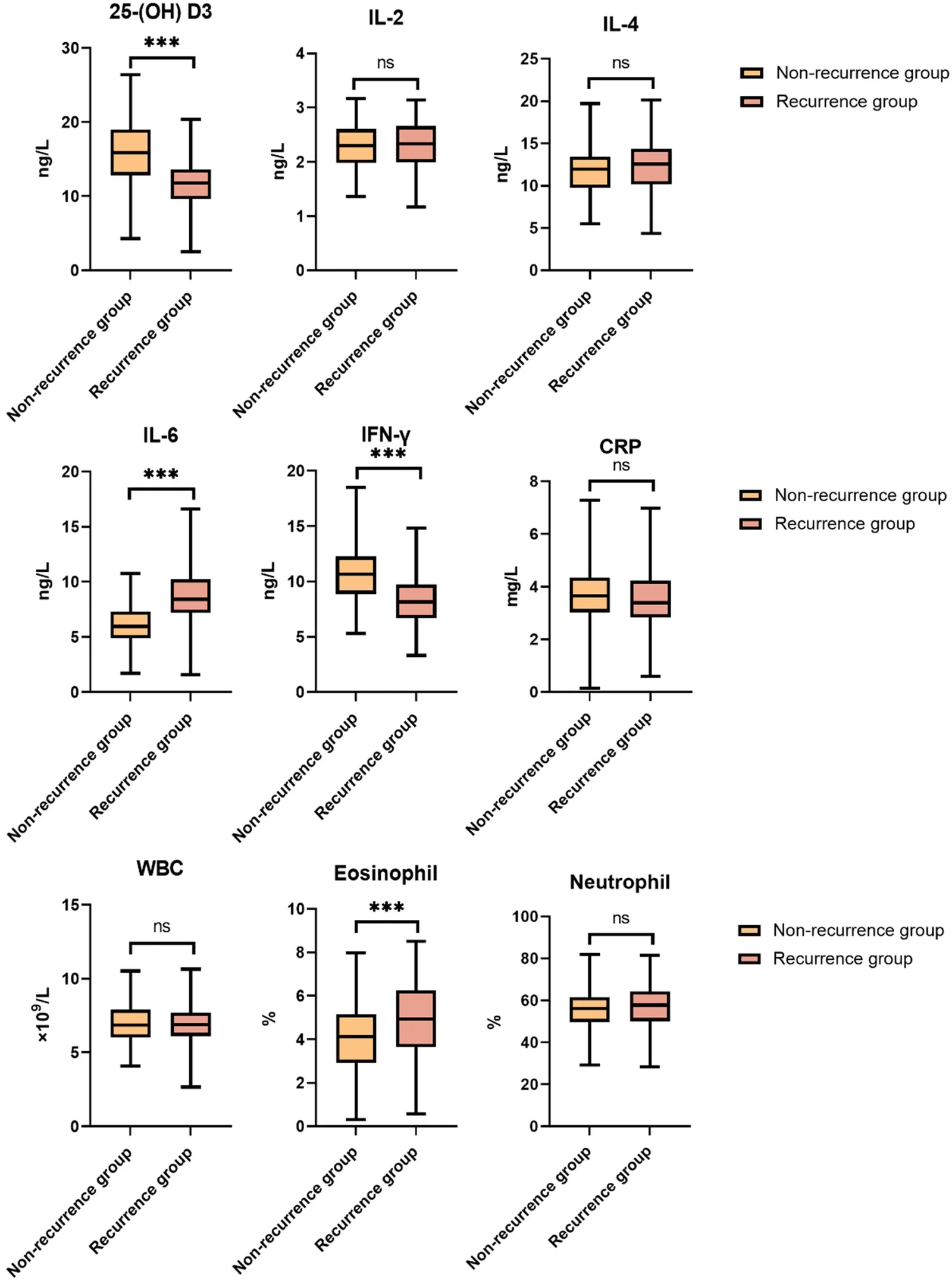
Serum markers between two groups in the training set 25-(OH) D3, 25-hydroxyvitamin D3; IL, interleukin; IFN-*γ*, interferon-*γ*; CRP, C-reactive protein; WBC, white blood cell count; ns, no statistically significant difference; ***, *P* < 0.001.

Serum marker comparisons for the training cohort are presented in Figure 1. IL-2, IL-4, CRP, WBC, and neutrophil percentage did not differ significantly between groups (all *P* > 0.05). Children who subsequently experienced recurrence displayed substantially lower 25-(OH) D3 and IFN-*γ* concentrations than those without recurrence (both *P* < 0.001), whereas IL-6 levels and eosinophil percentages were significantly elevated in the recurrence group (both *P* < 0.001).

#### 3.1.3 Variable filtering

Univariate and multivariate logistic regression results are displayed in Table 2. In univariable analysis, age (OR = 0.364, *P* < 0.001), lower 25-(OH) D3 (OR = 0.738, *P* < 0.001), reduced IFN-*γ* (OR = 0.581, *P* < 0.001), elevated IL-6 (OR = 1.561, *P* < 0.001), and higher eosinophil percentage (OR = 1.283, *P* < 0.001) were associated with lower or higher odds of recurrence, respectively, along with several clinical factors including passive smoking, prolonged hospital stay, history of allergies, and family history of asthma (all *P* < 0.05).

**Table 2:** Univariate and multivariate logistic regression analysis of recurrent wheezing risk within one year after initial hospitalization for RSV LRTI in preschool children.

Variables	Univariate analysis		Multivariate analysis	
	OR (95% CI)	P	OR (95% CI)	P
Age	0.364 (0.234–0.554)	<0.001	0.398 (0.205–0.773)	0.007
Preterm infant	1.670 (1.035–2.719)	0.037	1.472 (0.658–3.292)	0.346
History of allergies	1.846 (1.121–3.055)	0.016	2.135 (0.911–5.000)	0.081
Family history of asthma	1.978 (1.247–3.158)	0.004	1.648 (0.756–3.590)	0.209
Exposure to passive smoking	1.704 (1.061–2.760)	0.029	2.802 (1.239–6.336)	0.013
Length of hospital stay	1.289 (1.167–1.432)	<0.001	1.264 (1.083–1.475)	0.003
25-(OH) D3	0.738 (0.678–0.796)	<0.001	0.766 (0.690–0.850)	<0.001
IL-6	1.561 (1.386–1.777)	<0.001	1.591 (1.338–1.891)	<0.001
IFN-*γ*	0.581 (0.502–0.664)	<0.001	0.563 (0.460–0.687)	<0.001
Eosinophil	1.283 (1.122–1.476)	<0.001	1.398 (1.112–1.760)	0.004

RSV, respiratory syncytial virus; LRTI, lower respiratory tract infection; OR, odds ratio; CI, confidence interval; 25-(OH) D3, 25-hydroxyvitamin D3; IL, interleukin; IFN-γ, interferon-γ.

In multivariable analysis, after adjustment for all candidate variables, preterm birth, history of allergies, and family history of asthma were no longer statistically significant (all *P* > 0.05). Age (OR = 0.398, *P* = 0.007), passive smoking (OR = 2.802, *P* = 0.013), longer hospital stay (OR = 1.264, *P* = 0.003), lower 25-(OH) D3 (OR = 0.766, *P* < 0.001), higher IL-6 (OR = 1.591, *P* < 0.001), lower IFN-*γ* (OR = 0.563, *P* < 0.001), and higher eosinophil percentage (OR = 1.398, *P* = 0.004) remained as independent predictors.

The discriminatory performance of individual predictors is illustrated by the ROC analysis in Table 3. Age, preterm birth, history of allergies, family history of asthma, passive smoking exposure, length of hospital stay, and eosinophil percentage all exhibited relatively low AUC values, indicating limited standalone predictive accuracy. In contrast, 25-(OH) D3 (AUC = 0.785), IL-6 (AUC = 0.777), and IFN-*γ* (AUC = 0.787) demonstrated good discriminatory ability. These results suggest that serum markers possess superior predictive value for recurrent wheezing risk compared with clinical and demographic factors.

**Table 3:** ROC analysis of recurrent wheezing risk within one year after initial hospitalization for RSV LRTI in preschool children.

Variables	Best threshold	Sensitivities	Specificities	AUC	Youden index	F1 score
Age	4.575	0.741	0.535	0.666	0.276	0.314
Preterm infant	0.500	0.425	0.693	0.559	0.118	0.516
History of allergies	0.500	0.759	0.370	0.564	0.129	0.684
Family history of asthma	0.500	0.586	0.583	0.584	0.169	0.620
Exposure to passive smoking	0.500	0.448	0.677	0.563	0.125	0.532
Length of hospital stay	8.375	0.454	0.858	0.675	0.312	0.583
25-(OH) D3	15.060	0.885	0.598	0.785	0.483	0.148
IL-6	7.165	0.753	0.732	0.777	0.485	0.773
IFN-γ	10.190	0.845	0.575	0.787	0.420	0.197
Eosinophil	5.170	0.448	0.756	0.619	0.204	0.551

RSV, respiratory syncytial virus; LRTI, lower respiratory tract infection; ROC, receiver operating characteristic; AUC, area under the curve; 25-(OH) D3, 25-hydroxyvitamin D3; IL, interleukin; IFN-γ, interferon-γ.

#### 3.1.4 Model construction

**Figure 2:**
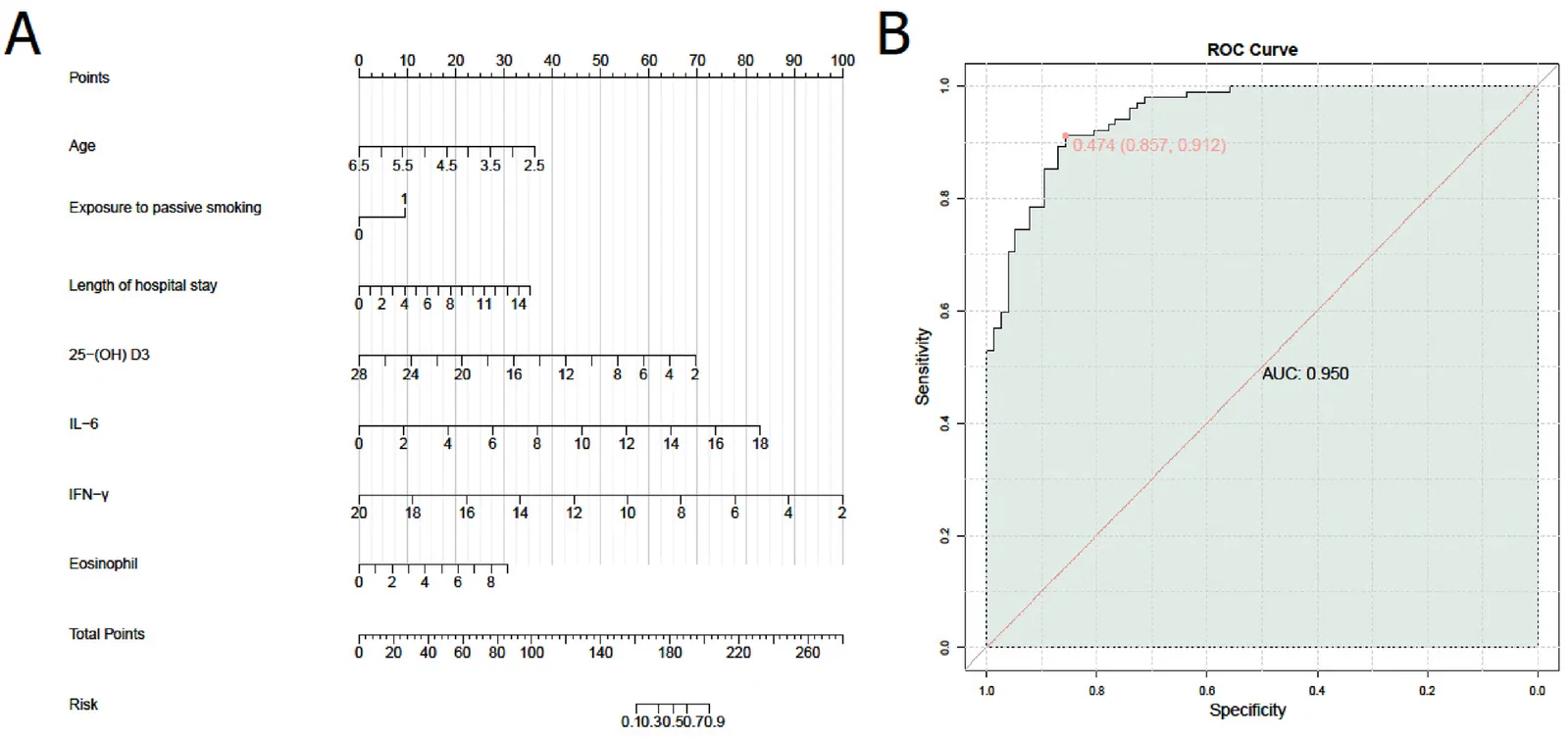
Construction of a predictive model for recurrent wheezing risk within one year after initial hospitalization for RSV LRTI in preschool children in the training set **(A)** nomogram; **(B)** ROC curve RSV, respiratory syncytial virus; LRTI, lower respiratory tract infection; ROC, receiver operating characteristic; AUC, area under the curve; 25-(OH) D3, 25-hydroxyvitamin D3; IL, interleukin; IFN-*γ*, interferon-*γ.*

Figure 2A presents a nomogram for predicting the risk of a specific clinical outcome, incorporating multiple variables including age, exposure to passive smoking, length of hospital stay, serum 25-(OH) D3 levels, IL-6 concentration, IFN-*γ* level, and eosinophil percentage. Each variable is assigned points based on its value, which are summed to calculate a total score corresponding to an individual's predicted risk level ranging from 0.1 to 0.9. Figure 2B displays the ROC curve for this predictive model, demonstrating excellent discriminative ability with an area under the curve (AUC) of 0.950. The optimal cutoff point at 0.631 provides a sensitivity of 0.912 and specificity of 0.857, indicating high accuracy in distinguishing between outcomes. Together, these results suggest that the model has strong predictive performance and clinical utility in risk stratification.

### 3.2 Validation set

#### 3.2.1 Demographic and clinical factors in the validation set

The demographic and clinical characteristics of the validation cohort are summarized in Table 4. Gender, BMI, and place of residence did not differ significantly between groups (all *P* > 0.05). Children who experienced recurrence were significantly younger (*P* = 0.006) and had markedly higher proportions of preterm birth (*P* = 0.029), personal history of allergies (*P* = 0.015), family history of asthma (*P* = 0.005), and exposure to passive smoking (*P* = 0.015) compared with those who remained recurrence-free. The length of hospital stay was also significantly prolonged in the recurrence group (*P* < 0.001). These findings confirm that younger age, preterm delivery, allergic predisposition, familial asthma history, passive smoking, and longer initial hospitalization are consistently associated with recurrent wheezing risk across both training and validation cohorts.

**Table 4:** Demographic and clinical factors in the validation set.

Variables	Non-recurrence group (*n* = 77)	Recurrence group (*n* = 102)	t/χ^2^	P
Gender, n(%)			0.020	0.888
Male	46 (59.74%)	62 (60.78%)		
Female	31 (40.26%)	40 (39.22%)		
Age (years)	4.57 ± 0.62	4.32 ± 0.58	2.793	0.006
BMI (kg/m^2^)	16.49 ± 2.43	16.21 ± 2.64	0.714	0.476
Place of residence, n(%)			0.000	0.996
Urban	43 (55.84%)	57 (55.88%)		
Country	34 (44.16%)	45 (44.12%)		
Preterm infant, n(%)			4.775	0.029
Yes	21 (27.27%)	44 (43.14%)		
No	56 (72.73%)	58 (56.86%)		
History of allergies, n(%)			5.876	0.015
Yes	45 (58.44%)	77 (75.49%)		
No	32 (41.56%)	25 (24.51%)		
Family history of asthma, n(%)			7.859	0.005
Yes	29 (37.66%)	60 (58.82%)		
No	48 (62.34%)	42 (41.18%)		
Exposure to passive smoking, n(%)			5.953	0.015
Yes	21 (27.27%)	46 (45.10%)		
No	56 (72.73%)	56 (54.90%)		
Length of hospital stay (d)	6.31 ± 2.18	7.78 ± 2.65	3.960	<0.001

BMI, body mass index.

#### 3.2.2 Serum markers in the validation set

Serum marker comparisons for the validation cohort are presented in Table 5. IL-2, IL-4, CRP, WBC, and neutrophil percentage did not differ significantly between groups (all *P* > 0.05). Children in the recurrence group had substantially lower 25-(OH) D3 and IFN-*γ* concentrations than those without recurrence (both *P* < 0.001), while IL-6 levels (*P* < 0.001) and eosinophil percentages (*P* = 0.004) were significantly elevated. These results confirm the training set findings, reinforcing that a distinct immunological profile combining vitamin D deficiency, reduced IFN-*γ*, heightened IL-6, and eosinophilic inflammation is robustly associated with recurrent wheezing risk after RSV lower respiratory tract infection.

**Table 5:** Serum markers between two groups in the validation set.

Variables	Non-recurrence group (*n* = 77)	Recurrence group (*n* = 102)	t	P
25-(OH) D3 (ng/L)	16.03 ± 4.51	11.56 ± 3.34	7.322	< 0.001
IL-2 (ng/L)	2.29 ± 0.43	2.31 ± 0.45	0.201	0.841
IL-4 (ng/L)	11.91 ± 3.08	12.45 ± 3.13	1.160	0.248
IL-6 (ng/L)	6.21 ± 1.93	8.56 ± 2.68	6.788	< 0.001
IFN-γ (ng/L)	10.62 ± 2.24	8.28 ± 1.95	7.463	< 0.001
CRP (mg/L)	3.74 ± 1.05	3.54 ± 1.08	1.231	0.220
WBC ( × 109/L)	6.95 ± 1.36	6.91 ± 1.25	0.223	0.824
Eosinophil (%)	4.17 ± 1.65	4.96 ± 1.87	2.946	0.004
Neutrophil (%)	56.22 ± 10.31	57.32 ± 10.66	0.690	0.491

25-(OH) D3, 25-hydroxyvitamin D3; IL, interleukin; IFN-γ, interferon-γ; CRP, C-reactive protein; WBC, white blood cell count.

#### 3.2.4 Model validation

**Figure 3:**
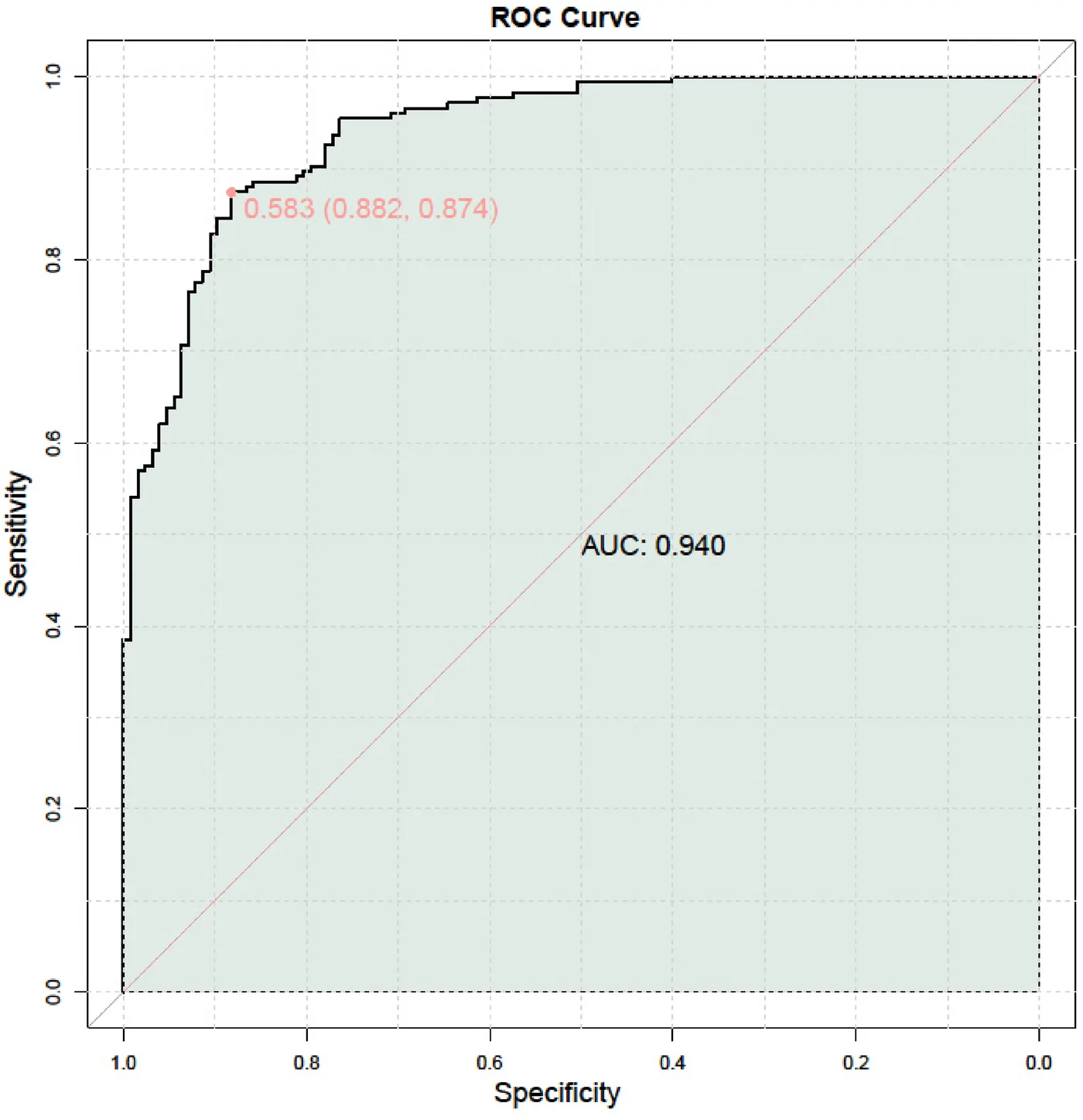
ROC curve of the predictive model for recurrent wheezing risk within one year after initial hospitalization for RSV LRTI in preschool children in the validation set RSV, respiratory syncytial virus; LRTI, lower respiratory tract infection; ROC, receiver operating characteristic; AUC, area under the curve.

Figure 3 presents the ROC curve for a predictive model assessing the risk of recurrent wheezing within one year after initial hospitalization for RSV LRTI in preschool children, evaluated in the validation cohort. The AUC is 0.940, indicating excellent discriminative ability of the model in distinguishing between children who will and will not experience recurrent wheezing. The curve demonstrates high sensitivity across a wide range of specificity values, with an optimal cutoff point at 0.583 corresponding to a sensitivity of 0.874 and specificity of 0.882, suggesting strong clinical utility for risk stratification. These results confirm the robustness and accuracy of the predictive model in the validation set, supporting its potential application in identifying high-risk children for targeted interventions.

## 4 Discussion

This study explores the construction and validation of a predictive model for assessing the risk of recurrent wheezing within one year after the first hospitalization for RSV LRTI in preschool children. The results indicate that a multivariate model integrating clinical characteristics, environmental exposures, and immune markers has excellent discriminatory ability and can provide a tool for early identification of high-risk children.

Younger age emerged as an independent predictor of recurrence, consistent with the well-recognized vulnerability of the developing respiratory and immune systems (16). Infants and younger preschool children exhibit narrower airways, immature antiviral defenses, and a propensity toward type 2-skewed immune responses after RSV infection, all of which fuel persistent wheezing (17). This finding aligns with numerous birth cohort studies reporting that early-life severe RSV bronchiolitis is more strongly associated with subsequent recurrent wheezing or asthma in children infected during the first years of life (18). In our multivariable model, preterm birth, a personal history of allergies, and a family history of asthma were significant in univariable analyses but did not retain independence. This pattern suggests that the effects of these traditional atopic and perinatal risks on post-RSV recurrent wheezing are largely mediated through downstream biological pathways captured by the biomarkers and lung function parameters included in the model. For example, the propensity conferred by an allergic predisposition or familial asthma heritage may be expressed through elevated eosinophilic inflammation or disturbed cytokine networks, and preterm-related pulmonary vulnerability may be reflected in reduced forced expiratory ratios (19). These observations resonate with studies showing that atopic family history loses predictive power when inflammatory markers are concurrently considered, underscoring the importance of measuring proximal immune mediators rather than relying solely on clinical history (20).

Passive smoking exposure was independently associated with a substantially elevated recurrence risk, a finding corroborating a wealth of pediatric literature linking environmental tobacco smoke to persistent airway injury, oxidative stress, and skewed Th2 immunity (21). Environmental tobacco smoke impairs mucociliary clearance, enhances epithelial permeability, and amplifies allergen sensitization, thereby establishing a chronic inflammatory milieu that perpetuates post-viral wheeze (22). The prolonged length of hospital stay, a proxy for the severity of the acute RSV insult, retained its independent predictive value. Extended hospitalization reflects more intense lower airway inflammation and epithelial damage, likely delaying repair and fostering airway remodeling that precipitates later wheezing exacerbations. Previous investigations have similarly identified initial disease severity, measured by length of hospitalization or need for intensive care, as a harbinger of recurrent post-bronchiolitis wheezing (23).

Among serum markers, reduced 25-(OH) D3 and IFN-*γ* levels, together with elevated IL-6 and eosinophil percentages, constituted a distinct immunologic signature independently associated with recurrence. The strong link between vitamin D insufficiency and post-RSV wheezing mirrors experimental and epidemiological data demonstrating that vitamin D supports antimicrobial peptide production, promotes regulatory T-cell expansion, and restrains Th2-driven eosinophilic inflammation, while its deficiency facilitates unchecked viral replication and a subsequent wheezing phenotype (24, 25). Depressed IFN-*γ* responses in the recurrence group imply a defective Th1 antiviral defense, tilting the immune balance toward an unrestrained Th2 milieu that favors IgE production and eosinophilic infiltration, a mechanism long postulated in virus-induced wheezing inception (26, 27). Several investigators have reported similar shifts in INF-*γ* production after RSV infection in children who later develop recurrent wheeze (28, 29). Simultaneously, heightened IL-6 appears to perpetuate airway injury and remodeling by mobilizing neutrophils, inducing mucus metaplasia, and stimulating fibroblast activity, thereby bridging acute inflammation with chronic airway changes. The independent contribution of eosinophil percentage extends well-established evidence that eosinophilic airway inflammation is a hallmark of recurrent wheezing (30). The superior discriminative capacity of these immune-functional markers over demographic factors alone, as reflected by ROC analyses, reinforces the notion that biological phenotyping substantially refines risk estimation.

By integrating the above predictors into a single nomogram, the model substantially outperformed any individual variable, achieving high discriminative accuracy in both cohorts. The predictive superiority of a multimodal instrument is in keeping with contemporary precision medicine approaches that recognize the multifactorial nature of wheezing disorders. Other risk scores developed for post-bronchiolitis wheeze have often relied predominantly on clinical criteria; the current model extends this strategy by incorporating objective laboratory measures that reveal the underlying immunological and functional status. The robust performance upon internal validation suggests reproducibility, though the single-center retrospective design inevitably constrains generalizability.

Beyond risk stratification, the present nomogram offers concrete guidance for initiating targeted interventions in clinical practice. Once a child is identified as high-risk (e.g., predicted probability > 0.6–0.7), the model's component variables directly inform actionable steps: (1) Vitamin D supplementation—given that low 25-(OH) D3 is an independent predictor, children with borderline or deficient levels at admission could be prescribed age-appropriate vitamin D replacement therapy before discharge, potentially modulating the Th2-skewed immune response and reducing eosinophilic inflammation; (2) Smoking cessation counseling—the strong independent association of passive smoking with recurrence provides an evidence-based impetus for pediatricians to deliver structured tobacco-exposure-reduction advice to caregivers at the bedside, with referral to smoking-cessation programs where available; (3) Enhanced surveillance—children with prolonged hospital stays (reflecting greater acute severity) or elevated IL-6/eosinophil profiles may benefit from scheduled follow-up visits at 1–3 months post-discharge rather than routine 6-month intervals, enabling early detection and prompt treatment of recurrent episodes; and (4) Personalized anti-inflammatory strategies—identifying a high IL-6/low IFN-*γ* immune signature could, in future, support the selection of children who might benefit from immunomodulatory or biologic therapies targeting type 2 inflammation. By translating statistical predictions into these specific, modifiable targets, the nomogram bridges the gap between prognostic modeling and bedside clinical action.

This study has several limitations. First, the retrospective, single-center design entails inherent selection bias and restricts the generalizability of the model. All included patients were from a single tertiary hospital in Shandong Province, China, and therefore may not capture geographic or demographic variations in RSV epidemiology, vitamin D status, or environmental exposures across different regions. Multi-center prospective studies involving diverse geographic regions and demographic populations are urgently needed to externally validate the model's discriminative performance, recalibrate its predictive thresholds, and evaluate its clinical utility in varied healthcare settings. Second, the follow-up duration of one year, although clinically relevant for early post-infection risk assessment, may not fully capture the long-term trajectory of wheezing, as some children may manifest recurrences beyond this window or outgrow early wheezing. Third, biomarkers were measured at a single time point during the index hospitalization, which precludes assessment of dynamic changes that might more accurately reflect evolving risk. Fourth, the inclusion of 25-(OH) D3, IL-6, IFN-*γ*, and other cytokine markers — which are not routinely measured in most clinical settings for RSV LRTI — may limit the immediate bedside applicability of the full model in resource-constrained or lower-level hospitals. Future work should explore simplified versions using only routinely available parameters (e.g., eosinophil percentage, length of stay, age, passive smoking) or externally validate the model in centers with similar testing capabilities. Finally, future prospective studies should also examine whether interventions targeting modifiable predictors—such as vitamin D supplementation, tobacco smoke reduction, or anti-inflammatory therapies tailored to immune profiles—can mitigate the risk of recurrent wheezing in high-risk children identified by this model.

## 5 Conclusion

This study developed and internally validated a nomogram integrating age, passive smoking exposure, length of hospital stay, serum 25-(OH) D3, IL-6, IFN-*γ*, and eosinophil percentage, which demonstrated excellent discriminative performance for predicting recurrent wheezing within one year after initial RSV lower respiratory tract infection in preschool children, offering a practical bedside tool for early risk stratification and personalized management.

## Data Availability

The raw data supporting the conclusions of this article will be made available by the authors, without undue reservation.
